# Sedative and Analgesic Effects of Entonox Gas Compared with Midazolam and Fentanyl in Synchronized Cardioversion

**DOI:** 10.1155/2015/798478

**Published:** 2015-10-20

**Authors:** Kambiz Masoumi, Arash Forouzan, Sina Saghari, Maryam Feli, Ali Reza Sattari, Ali Asgari Darian

**Affiliations:** ^1^Department of Emergency Medicine, Ahvaz Jundishapur University of Medical Sciences, Ahvaz 6193673166, Iran; ^2^Department of Emergency Medicine, Kerman University of Medical Sciences, Kerman 7616914115, Iran

## Abstract

The purpose of this study was to determine if the Entonox gas could cause adequate analgesic and sedative effects in patients who need cardioversion. In this randomized not blinded clinical trial, the sedative and analgesic effects of midazolam and fentanyl were compared with Entonox. Eligible patients who need synchronized cardioversion because of dysrhythmia were randomly divided into two groups. The first group received intravenous midazolam and fentanyl; the second group received Entonox through a blower-dependent mask. Onset and end of sedation, sedation level, and pain score were recorded. There were nonsignificant differences between the two groups (22 patients in each group) regarding age, gender, weight, sedation level, and frequency and level of shock. The pain score recorded in the first group was 5.05 ± 1.32, and 3.9 ± 0.7 in the second group (*P* = 0.002). Furthermore, sedation duration and time to full recovery consciousness were shorter in the second group (*P* < 0.001). In the first group, seven patients needed additional doses to induce and maintain sedation. In addition, as a result of apnoea, four patients required airway support. None of them occurred in the second group. Entonox is a suitable medication in rapid cardioversion, as it has minimal side effects and adequate analgesic and sedative effects.

## 1. Introduction

Sedation and analgesia in medical procedures refer to the use of sedatives, analgesics, and amnetics, to relieve the pain and anxiety caused by diagnostic and therapeutic procedures carried out in a variety of circumstances. Emergency physicians develop the skills to carry out procedures such as resuscitation, vascular access, cardioversion, and advanced airway management, which require sedation and analgesia [1]. Moreover, sedation and analgesia lead to improved patient care and satisfaction, which, ultimately, facilitates diagnostic and therapeutic procedures [2].

There are many medications used for sedation and analgesia that have the potential to suppress respiratory, cardiovascular, and central nervous systems [3].

Cardioversion is referred to as the passing of a direct electrical current (shock) through the chest wall or directly over the ventricle, in order to produce rapid normalization of the cardiac conduction pattern. The synchronized shock is given while the QRS wave in the absolute refractory period of an ECG, concurrently at peak R wave [4]. Cardioversion is one of those procedures that can be intensely frightening and painful. Thus, the patient should be adequately sedated before cardioversion is performed. Patients that have not received adequate levels of sedatives and analgesics may experience severe pain and fright [5]. There are numerous intravenous drugs available for inducing sedation and analgesia prior to cardioversion, including Etomidate, fentanyl, midazolam, Propofol, Thiopental, and Methohexital [4]. In high pain and high anxiety procedures such as cardioversion, the best choice for sedation and analgesia is intravenous midazolam-fentanyl in adults [2].

Midazolam is probably the most common drug used for sedation and analgesia before cardioversion, and anesthesia is induced through intravenous administration of 0.15 mg/kg, or a minimum of 5 mg for average-size adults, as it acts within 2 minutes, and it also has long-term effects when compared to other drugs. The addition of a small dose of fentanyl (1.5 mcg/kg) may increase the depth of sedation. However, fentanyl can also lead to respiratory suppression [4].

Nitrous oxide (N_2_O) is a gas, which, combined with oxygen, creates an excellent analgesia for sedation required in a number of medical procedures. This mixture spreads rapidly in the alveoli and it has a rapid and predictable onset time of about 1 to 2 minutes, with a rapid clearance time of about 3 to 5 minutes. This gas is used in different ratios (30% to 70%) with oxygen and administered through a mask or a unilateral oral piece held by the patient [2]. The amount of gas inhaled is controlled by the patient. Thus, inhalation stops when the gas takes effect, and it creates sedation at the minimum required dose. Despite its safety and lack of side effects, nitrous oxide may fail to ensure adequate analgesia, but with continuous administration through a mask or the use of higher percentages deep sedation can be achieved [6].

Entonox is readily available in capsules containing equal proportions of 50% nitrous oxide and 50% oxygen. In previous studies, the sedative effect of Entonox has been compared with other sedatives in shoulder dislocation, colonoscopy, and urologic procedures such as extracorporeal shock wave lithotripsy (ESWL) [7–9].

This study aimed to compare analgesic and sedative effects of Entonox compared with midazolam and fentanyl combination in synchronized cardioversion.

## 2. Material and Methods

### 2.1. Trial Design

This randomized, not blind clinical trial was conducted on patients admitted to the Emergency Department (ED) due to symptomatic dysrhythmia. Before commencement of the study, the Code of Ethics numbered ETH-534 was obtained from the Ethic Committee of Ahvaz Jundishapur University of Medical Sciences, and every stage of the study was designed and implemented according to the Helsinki Declaration 1975. Written consent was obtained from all participating patients, and confidentiality of patient's personal details was maintained. Patients were not charged for their participation in this study, and all necessary disposable and nondisposable materials were procured by the researchers. This study was registered at Iranian Clinical Trials Registration Centre, and the relevant code was obtained.

### 2.2. Participants

#### 2.2.1. Inclusion and Exclusion Criteria

Inclusion criteria were patients over 18 years of age of both sexes that required synchronized cardioversion presenting to ED of Imam Khomeini Hospital, Ahvaz, Iran, from April 2013 to December 2014. Exclusion criteria were drug sensitivity to nitrous oxide compounds, pneumothorax, small bowel obstruction, chronic obstructive pulmonary diseases, head trauma associated with altered mental status, maxillofacial trauma, air emboli, otitis media and collection, and pregnancy.

#### 2.2.2. Interventions

Initially, an ECG was taken from fully conscious patients who had been admitted to the Emergency Department with complaints such as chest pain, dyspnea, vertigo, and palpitations. When tachydysrhythmia associated with symptoms requiring cardioversion (unstable tachydysrhythmia with palpable pulse regarding patient conditions) was detected, patients were transferred to the Cardiopulmonary Resuscitation Unit and they underwent cardiac monitoring, pulse oximetry, and obtaining capnography. Patients' vital signs were taken and recorded. After administering oxygen and peripheral vascular access, a brief and targeted medical history was taken and recorded from accompanying person or patient if his/her condition permits including previous diseases, medication used, self-reported age, and weight of each patient. Next, the patient's written consent was obtained after a full explanation of the study was given to every patient or their family. Patients in our study had never been fasted.

In the first group, sedation was achieved using a 30-second intravenous administration of midazolam (0.15 mg/kg) [Midazolex 5, Exir Co., Iran; midazolam 5 mg/mL] and fentanyl (1.5 mcg/kg) [Feniject 0.5, Aburaihan Co., Iran, fentanyl 0.5 mg/10 mL].

Once the patient had reached a sedation level of −3 to −4 based on the Richmond Agitation Sedation Scale [10, 11], according to the patient's cardiac rhythm, the required synchronized electrical shock was given. Afterwards, if the tachydysrhythmia was not controlled, after checking the patient's sedation, further shocks were administered if the sedation level persisted. Dosage was increased if the intended sedation level was not achieved. Minimum biphasic shock administered was 50 joules and maximum 200 joules. In this group, the number of shocks administered varied from one to three. Timing of sedative administration, onset of appropriate sedation level, and return to full recovery of consciousness were recorded.

**Figure 1:**
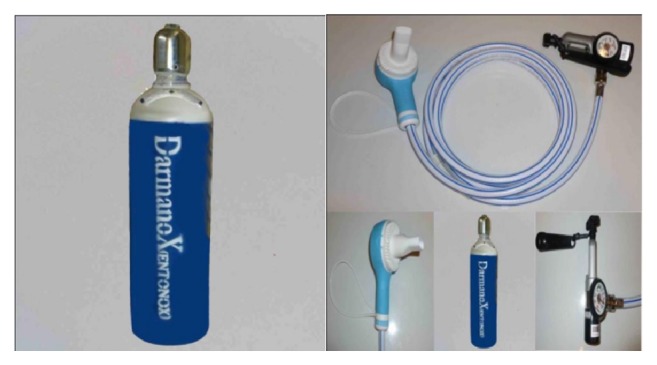
Darmanox capsule and giving set which were used in this study.

In the second group, Entonox gas [Darmanox 20-litre cylinder: Darmangaz Company, registration number 47062, Iran; giving set: BPR medical company, UK, 50% nitrous oxide and 50% oxygen mixed] “Figure 1” was used to induce sedation by a patient-controlled analgesia pump with a handset. If the patient condition permitted, the method of gas administration was first explained to the patient, and he/she was asked to place the gas inhalation mouthpiece into his/her mouth on their own and inhale the gas by sucking, according to the instructions. Entonox was administered through a mouthpiece connected to an Entonox cylinder. This mouthpiece has a one-way demand valve system, which is operated by the act of inhalation of the patient and closes down when the patient ceases to inhale. Once an appropriate level of consciousness was reached and after spontaneously dropping the mouthpiece from the patient's mouth, a synchronized electrical shock was administered according to the patient's cardiac rhythm. After controlling the sedation level, if the tachydysrhythmia was not controlled, a shock was given at a higher level.

In both groups, the patients' vital signs, respiratory efforts, and end-tidal CO_2_ were carefully monitored during sedation and reduced consciousness periods.

### 2.3. Outcomes

#### 2.3.1. Primary Outcomes

The primary end-point measured was the degree of pain experienced by the patient during the procedure and assessed on a validated 100 mm VAS. After returning to their initial consciousness level, the patients were asked about their level of pain and this was recorded according to the visual analogue scale (VAS). VAS is a 10 cm ruler with 100 divisions, and the patients were given explanation as follows: “One side of the ruler indicates a lack of any sense of pain and the other side indicates extreme pain.” Patients were then asked to mark the level of pain they experienced during shock on the ruler. After marking the other side of the ruler, the pain level was read and recorded in numbers from 0 to 10, with one decimal point. When the effect of the sedatives had worn off and the patient had returned to initial consciousness level, they were monitored for at least an hour, in order to control and assess any potential complications.

#### 2.3.2. Secondary Outcomes

The secondary end-points measured included sedation duration, time to full recovery consciousness, and need of additional doses to induce and maintain sedation.

#### 2.3.3. Sample Size Calculation

To compare the mean differences in pain and sedation levels in the patients who received Entonox or midazolam with fentanyl, using NCSS software, with 90% power, 95% confidence interval, variance of 1, and minimum pain reduction of 2 units (primary outcome measure), the sample size required for each group was 22 [12].

#### 2.3.4. Randomization and Allocation Concealment

The patients were randomized by using simple randomization method. The assignments were held centrally in sequentially numbered, opaque, and sealed envelopes, and the envelopes were opened sequentially. The randomization sequence was created by a person not involved in this study. Both medical staffs and patients were aware of treatment modality after each envelop was opened.

### 2.4. Statistical Analysis

To analyse and compare pain levels and the onset and end of sedation in the two groups (patients that received Entonox versus patients that received midazolam with fentanyl), an independent *t*-test was used. In addition, to analyse qualitative parameters such as gender, chi-square, comparison of incidence rate, need for airway management, and higher doses of sedative, Fisher's exact test was used.

## 3. Results

At the outset of the study, 103 patients were candidates for cardioversion, of whom 39 did not require electrical shock and underwent medical cardioversion based on clinical judgments of physicians. There were 20 patients who did not give their consent to take part in the study.

**Figure 2:**
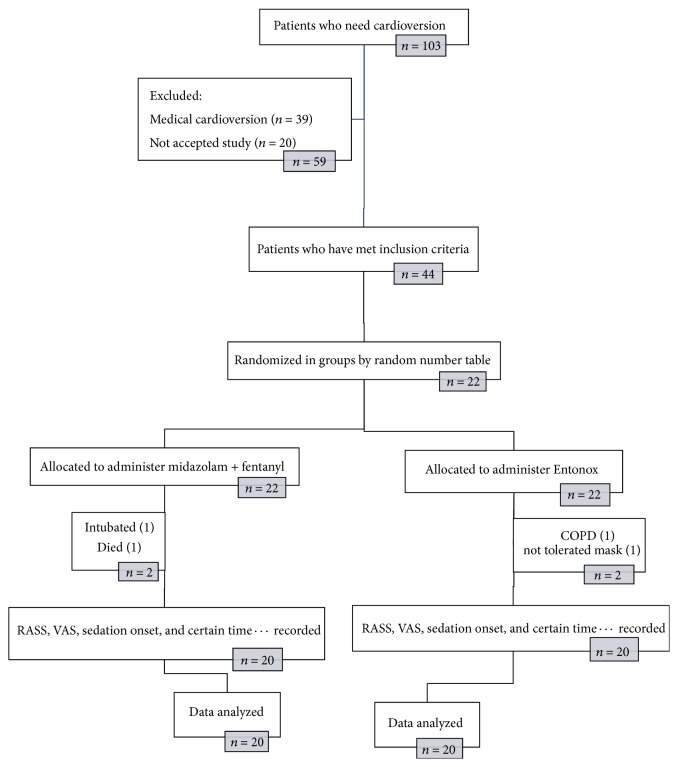
Randomized controlled trial flowchart of patients' recruitment is shown.

In total, 44 patients met the study inclusion criteria. In the first group (midazolam with fentanyl), one patient died. He was a known case of IHD and DM and he died following hospitalization in CCU, probably because of his background disease, and another patient was excluded due to their need for intubation. In the second group (Entonox), one patient who had a history of chronic obstructive pulmonary disease and one because of inappropriate cooperation were excluded from study (Figure 2).

According to analysis of the data, no significant differences in terms of gender (*P* = 0.51), age (*P* = 0.4), weight (*P* = 0.42), or sedation level based on RASS (*P* = 0.48) were found between the two groups (Table 1).

Patients in both groups received shocks for cardioversion. The number of shocks and the total dose of shocks based on Jules were compared between two groups, and no significant differences were found (*P* = 0.34 and *P* = 0.35, resp.). The onset of sedation time in the first group (midazolam with fentanyl) was significantly higher (*P* = 0.028). Furthermore, the mean return time to full recovery of consciousness in the first group was 60 minutes longer on average than that found in the Entonox group (*P* < 0.001). Patients in the Entonox group had significantly lower pain score (based on VAS) than patients in midazolam and fentanyl group (*P* = 0.005) (Table 2).

In the first group, seven patients (33.3%) required higher doses of drug in order to achieve the required consciousness level, but, in the Entonox group, no patient required an additional dose or any other drug. In the first group, four patients (19%) developed transient apnea, and they required temporary airway management but one of them was intubated because of prolonged apnea and deterioration. In the Entonox group, no patient developed apnea, but two patients experienced transient headaches, one developed vertigo, and 3 had thirst and xerostomia.

## 4. Discussion

Since the delivery of cardioversion typically results in patient anxiety and discomfort, patients should be adequately sedated to ameliorate the effects of the procedure. Optimally, the sedation and analgesia required to promote patient satisfaction and safety need not require overt airway management beyond a simple face mask or nasal cannula.

Midazolam is a short-acting benzodiazepine, usually used for mild sedation [13]. A moderate to deep sedation can be achieved with the addition of an opioid, such as fentanyl. With simultaneous administration of midazolam and fentanyl, the risk of respiratory suppression increases. Furthermore, given the 60-minute half-life of midazolam and fentanyl, it is necessary to monitor the patient during and after medical procedures. Nitrous oxide is usually available in a 50-50 form with oxygen. The action duration of this mixture is approximately 15 to 20 minutes [14]. Entonox has few side effects and mild cardiac suppression [15].

Considering the potentially fatal consequences of tachydysrhythmia, quick action to restore cardiac rhythm can save a patient's life. Furthermore, ending these potentially dangerous rhythms by cardioversion is extremely painful and stressful. Thus, given the abovementioned issues, it is essential to use an appropriate technique that can rapidly induce sedation with the least complications and without the need for an airway management equipment.

It is noteworthy that if the patient is able to return fast to a normal level of consciousness, this will also prevent complications such as aspiration and reduce their hospital stay [14].

In this study, attempts were made to compare the advantages and disadvantages of using Entonox in sedation during cardioversion, with midazolam and fentanyl. The study was designed and conducted according to available information resources and initial assumptions.

In terms of personal details, such as age, gender, and weight, and also potential confounding factors, such as sedation level, and frequency and level of electrical shocks, the differences between the two groups were insignificant. The level of pain according to the VAS, return time to consciousness, and total sedation time in second group patients who were sedated with Entonox were significantly lower than those found in the first group. In the midazolam and fentanyl group, 4 patients required airway management due to transient apnea including intubation in one patient, and one patient died because of deterioration in clinical condition and pulmonary edema, while no patient developed apnea or death in the Entonox group; therefore, the incidence rate of apnea, according to Fisher's exact test, was significantly lower in the Entonox group. The blood/gas partition coefficient of nitrous oxide is 34 times greater than that of nitrogen. This differential solubility means that nitrous oxide can leave the bloodstream and enter air-filled cavities 34 times faster than nitrogen. As a result, nitrous oxide is contraindicated in patients in whom expansion of these air-filled cavities could compromise patient safety. This includes patients with* pneumothorax*, pulmonary blebs, air embolism,* bowel obstruction*, and those undergoing surgery of the middle ear. As we said, one patient was excluded from our study because of COPD. In a study conducted in 2008-2009 by Mahshidfar et al. in Iran, the sedative action of Entonox was compared with midazolam and fentanyl in shoulder dislocation reduction of 120 patients with anterior dislocation of shoulders, using the VAS measuring system. According to their results, Entonox was found to be inappropriate in shoulder dislocation reduction, and there was a significant difference in pain levels between the two groups (*P* < 0.0001) [7]. In this study, Entonox had a greater analgesic action, compared to midazolam plus fentanyl, and this effect seems to be due to its muscle relaxation properties, whereas midazolam was more effective in shoulder reduction procedures. In 2007, a study was conducted in Iran by Mazdak et al. to investigate the analgesic action of inhaling Entonox in performing extracorporeal shock wave lithotripsy (ESWL) in 150 patients. According to the results obtained, Entonox can be effective in producing rapid and adequate relief of pain with minimum complications in patients requiring ESWL (*P* = 0.001) [16].

Also, in a study conducted in 2005-2006 by Maslekar et al. in the Hull and Castle Hill Hospital in Nottingham, England, the sedative action of Entonox was compared with midazolam-fentanyl in 131 colonoscopy patients. Ultimately, it was concluded that Entonox has better analgesic properties compared to the other two drugs, and patients had shorter recovery times [16]. In addition, in a study by Young et al., conducted as a review of articles in the Medline site in England, inhaling Entonox was investigated in reducing pain in urology outpatient diagnostic and medical procedures. According to the results, Entonox induces appropriate and effective analgesia without complications in urological procedures [9].

In 2002, a study was conducted by Coll-Vinent et al. in the Emergency Department of Barcelona Hospital, Spain, on four different sedation techniques in cardioversion. In this study, 32 adult patients with stable hemodynamics underwent cardioversion in the Emergency Department. Of whom, 9 patients were sedated with Etomidate, 9 with Propofol, 8 with midazolam, and 6 with midazolam followed by Flumazenil. According to the results obtained, sedation was equally effective and appropriate in all four groups, and Propofol was tolerated best by the patients. Finally, the authors recommended that their study results be subject to further studies [12].

## 5. Conclusion

According to the results obtained and the statistical analyses conducted on the results, combined with the similarity of results from other studies, Entonox was found to be an appropriate sedative drug in cardioversion as it has minimum side effects and induces appropriate analgesia. Additionally, Entonox is also a suitable drug in terms of sedation time and medical facilities required, which can be used in circumstances where there is a need for rapid cardioversion without any health care provider who trained in primary and advanced airway managements.

Due to the different drug administration methods and a probable shortage of experienced personnel in airway management in different facilities who were familiar with both sedation and cardioversion methods, conducting a double-blind study proved to be impractical. For generalizability of our study results, more extensive studies on patients of different ethnic origins are recommended.

## Figures and Tables

**Table 1:** The comparison of Entonox group with midazolam and fentanyl group in terms of age, weight, and sedation level.

Variable	Entonox	Midazolam + fentanyl	*P* value
Gender^*∗*^			
Male	14 (63.6)	16 (72.7)	0.51
Female	8 (36.4)	6 (27.3)	
Age (mean ± SD)	52 ± 10.2	55.4 ± 15.6	0.4
Weight (mean ± SD)	68.8 ± 10.1	66.4 ± 9.7	0.42
RASS (mean ± SD)	−3.5 ± 0.5	−3.7 ± 0.8	0.48

^*∗*^Number (%).

**Table 2:** The comparison of Entonox group with midazolam and fentanyl group regarding the need for shock, sedative, and analgesic effects (VAS: visual analogue scale).

Shocks	Entonox	Midazolam + fentanyl	*P* value
Number of shocks^*∗*^	1.20 ± 0.52	1.36 ± 0.58	0.34
Jules of shocks^*∗*^	86.00 ± 33.62	96 ± 37.86	0.35
Sedation onset^*∗*^	11.65 ± 3.58	14.36 ± 4.08	0.028
Full consciousness recovery^*∗*^	15.00 ± 3.77	75.00 ± 30.3	<0.001
VAS score	3.09 ± 0.7	5.05 ± 1.32	0.002

^*∗*^Mean ± SD (standard deviation).
